# Evaluation of a novel algorithm for primary mass casualty triage by paramedics in a physician manned EMS system: a dummy based trial

**DOI:** 10.1186/s13049-014-0050-6

**Published:** 2014-08-28

**Authors:** Philipp Wolf, Marc Bigalke, Bernhard M Graf, Torsten Birkholz, Michael S Dittmar

**Affiliations:** 1 Department of Anaesthesiology, Regensburg University Medical Center, Franz-Josef-Strauss-Allee 11, Regensburg, 93053, Germany; 2 Department of Anaesthesiology, Asklepios Hospital Burglengenfeld, Dr.-Sauerbruch-Str. 1, Burglengenfeld, 93133, Germany; 3 Medical Director of Emergency Medical Services for the EMS area Amberg (ÄLRD Amberg), Gasfabrikstr. 11, Amberg, 92224, Germany

**Keywords:** Mass casualty incident, Primary triage, ASAV, Reliability, Time requirement

## Abstract

**Background:**

The Amberg-Schwandorf Algorithm for Primary Triage (ASAV) is a novel primary triage concept specifically for physician manned emergency medical services (EMS) systems. In this study, we determined the diagnostic reliability and the time requirements of ASAV triage.

**Methods:**

Seven hundred eighty triage runs performed by 76 trained EMS providers of varying professional qualification were included into the study. Patients were simulated using human dummies with written vital signs sheets. Triage results were compared to a standard solution, which was developed in a modified Delphi procedure. Test performance parameters (e.g. sensitivity, specificity, likelihood ratios (LR), under-triage, and over-triage) were calculated. Time measurements comprised the complete triage and tagging process and included the time span for walking to the subsequent patient. Results were compared to those published for mSTaRT. Additionally, a subgroup analysis was performed for employment status (career/volunteer), team qualification, and previous triage training.

**Results:**

For red patients, ASAV sensitivity was 87%, specificity 91%, positive LR 9.7, negative LR 0.139, over-triage 6%, and under-triage 10%. There were no significant differences related to mSTaRT. Per patient, ASAV triage required a mean of 35.4 sec (75^th^ percentile 46 sec, 90^th^ percentile 58 sec). Volunteers needed slightly more time to perform triage than EMS professionals. Previous mSTaRT training of the provider reduced under-triage significantly. There were significant differences in time requirements for triage depending on the expected triage category.

**Conclusions:**

The ASAV is a specific concept for primary triage in physician governed EMS systems. It may detect red patients reliably. The test performance criteria are comparable to that of mSTaRT, whereas ASAV triage might be accomplished slightly faster. From the data, there was no evidence for a clinically significant reliability difference between typical staffing of mobile intensive care units, patient transport ambulances, or disaster response volunteers. Up to now, there is no clinical validation of either triage concept. Therefore, reality based evaluation studies are needed.

## Abstract (de)

**German Abstract:**

**Hintergrund**

Der Amberg-Schwandorf-Algorithmus für die Vorsichtung (ASAV) ist ein neues Ablaufschema für die Vorsichtung durch Nicht-Ärzte beim Massenanfall von Verletzten und speziell für notarztbasierte Rettungsdienstsysteme entwickelt. In der vorliegenden Arbeit untersuchten wir die Testgütekriterien und den Zeitbedarf für die Vorsichtung mittels ASAV.

**Methoden**

Für die Studie wurden 780 Sichtungsvorgänge von 76 Probanden unterschiedlicher Qualifikation ausgewertet. Als Patienten wurden Dummies, ausgestattet mit einer schriftlichen Zustandsbeschreibung verwendet. Die Sichtungsergebnisse wurden mit einer Musterlösung verglichen, welche in einem modifizierten Delphi-Prozess erstellt wurden. Daraus wurden Testgütekriterien wie Sensitivität, Spezifität, Wahrscheinlichkeitsverhältnisse (LR), Über- und Untertriage berechnet. Die Zeitmessung umfasste den kompletten Sichtungs- und Sichtungskennzeichnungsvorgang einschließlich der Wegezeit zum nächsten Patienten. Die Ergebnisse wurden zu den für mSTaRT veröffentlichten Daten verglichen. Zusätzlich wurde eine vordefinierte Subgruppenanalyse in Bezug auf Beschäftigungsverhältnis (Haupt- vs. Ehrenamt), Teamzusammensetzung und vorherige Sichtungsausbildung durchgeführt.

Bezogen auf die roten Patienten betrug die Sensitivität 87%, die Spezifität 91%, die positive LR 9,7, die negative LR 0,139, die Übertriage 6% und die Untertriage 10%. Signifikante Unterschiede zu mSTaRT konnten nicht nachgewiesen werden. Die Sichtung mittels ASAV benötigte durchschnittlich 35,4 Sek. pro Patient (75. Perzentil 46 Sek., 90. Perzentil 58 Sek.). Ehrenamtliche benötigten geringfügig länger für die Vorsichtung. Eine vorausgehende Ausbildung in einem anderen Sichtungskonzept reduzierte die Untertriage signifikant. Je nach erwarteter Sichtungskategorie unterschied sich der Zeitbedarf für die Vorsichtung signifikant.

**Schlussfolgerungen**

Der ASAV ist ein Konzept für die Vorsichtung speziell für notarztbasierte Rettungsdienstsysteme. Durch ASAV scheinen sich die roten Patienten verlässlich identifizieren zu lassen. Die Testgütekriterien sind vergleichbar mit den für mSTaRT publizierten Werten, wobei ASAV etwas weniger Zeit in Anspruch zu nehmen scheint. Unsere Daten ergaben keinen signifikanten Unterschied bei der Vorsichtungs-Reliabilität zwischen typischen Besatzungen von Rettungstransportwägen, Krankentransportwägen oder ehrenamtlichen Katastrophenschutzeinheiten. Gegenwärtig ist kein Vorsichtungsalgorithmus klinisch validiert. Daher sind weitere Untersuchungen an Realeinsätzen notwendig.

## Introduction

The global incidence of natural and technological disaster has been on a steady rise until around the year 2000. Since then, the numbers are slightly decreasing [1]. Despite ongoing efforts to implement major incidence registries [2], little is known on the incidence of sub-disaster mass casualty incidents (MCI). The notion that mortality in the MCI setting is still considerably higher than in individual cases [3] shows the necessity to further improve patient care in these situations.

In a physician manned emergency medical service (EMS) system, as present in many European countries [4], medical decision making is limited for non-physician EMS personnel. Triage algorithms provide a standardized and preliminary patient assessment and classification. In this context, many triage algorithms for mass casualty incidents (MCI) require the decision on withholding lifesaving interventions for presumably unsalvageable patients, as it is the case for START [5], Triage Sieve [6],[7], jumpSTaRT [8], mSTaRT [9], or SALT [10],[11]. Especially in the German physician governed EMS, such decisions are legally restricted to physicians. In addition, complex algorithms may be time consuming. Simplified algorithmic decision making and avoidance of withholding lifesaving interventions are therefore core requirements for primary triage by non-physician EMS personnel. Taking this into account, we adapted the mSTaRT primary triage algorithm [9] for the use in our physician based EMS system, resulting in the Amberg-Schwandorf Algorithm for Primary Triage (*Amberg-Schwandorf-Algorithmus für die Vorsichtung*, ASAV) [12]. Although results of primary EMS triage have a preliminary character, the algorithm must prove its reliability as a diagnostic test. In this study we report the evaluation of ASAV concerning its accuracy, reliability, and time requirements. Further, the influence of provider characteristics on the said parameters was analysed.

## Methods

After the positive vote of the ethics committee of the Regensburg University Medical Faculty (file reference 13-101-001), the study was performed during the ASAV implementation and education process.

### Study participants

Study participants were recruited from the professional EMS staff of the district of Schwandorf (Bavaria, Germany) as well as from volunteers of a local disaster relief organisation.

The study participants attended three hours of theoretical teaching (one hour on MCI tactics, and two hours on the application of ASAV), followed by one hour of team training in primary triage using the ASAV approach. Subsequently, data was collected during the practical certification exam.

### Dummies as patients

The triage was performed on dummies, which represented the MCI victims. The relevant vital data and other important information were written and posted on the dummies (dummy description cards). For practical training and data acquisition, 20 different individual cases were presented, respectively. The assignment of cases to either the training session or the certification exam, as well as the order of appearance, was randomised separately for each training session. For randomisation, a random number table was used [13].

**Figure 1:**
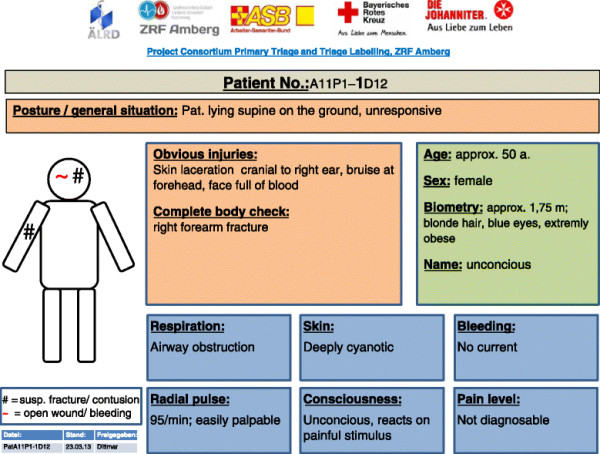
Exemplary illustration of one out of 40 different dummy description cards, which provided the patient data available to the triage personnel.

The dummy description cards presented information on the patient’s body posture, detectable external injuries, patient demographics, and signs and symptoms to the organ systems/functions respiration, skin, bleeding, pulse status, consciousness and pain level. In addition, the location of bleedings and suspected fractures were graphically displayed on a drawn body scheme (see Figure 1). No special incident scenario was provided to the study participant.

### Triage process

**Figure 2:**
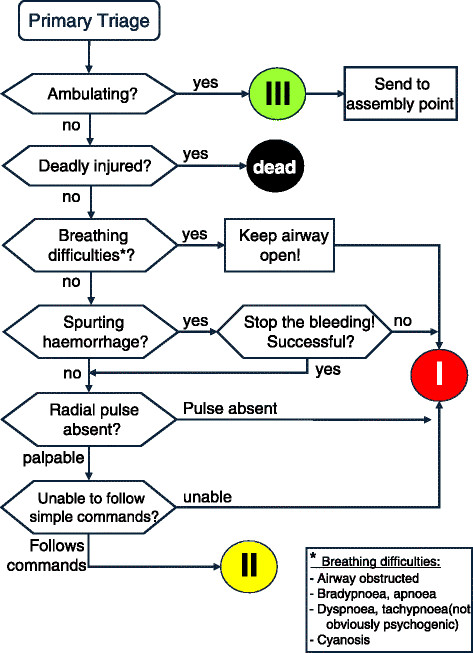
**The Amberg-Schwandorf Algorithm for Primary Triage (ASAV) (modified from Dittmar et al. [**[12]**], with permission).**

The ASAV algorithm is displayed in Figure 2. During the triage process, patients were assigned to one of the following triage categories: red (immediate treatment and/or transport), yellow (delayed treatment and transport), green (minor injuries), and black (dead). Red, yellow, and black patients were tagged according to their assigned triage category by coloured plastic bands. Green patients were not tagged.

Triage was performed in teams of two, with one member representing the team leader, who was responsible for the clinical assessment of patients, who made the triage decisions and applied the triage tags, and the triage assistant, who announced each triage step, and who took the task of documentation. After assessing ten patients, team roles were switched. The teams were trained to strictly follow the triage algorithm which was available to the triage assistant as a printed copy during the triage process.

During the lessons the participants were educated to apply oropharyngeal tubes for airway obstruction in unconscious patients and simple bleeding control measures for spurting haemorrhage, respectively. If those life saving interventions were deemed necessary by the triage team according to the descriptions on the dummy description cards, these were not executed on the dummy, but indicated by placing an oropharyngeal tube and/or a wound dressing on the patient dummy, respectively.

Each triage process was monitored by two study assistants, who documented the triage decisions and measured the time needed for each triage run as described below.

### Endpoints

Study endpoints were agreement of triage decisions with the standard solution in terms of triage category, and making a correct decision on lifesaving interventions (opening the airway or attempting bleeding control).

The standard solution was created in a modified Delphi approach. One medical director of EMS, a teaching paramedic, and an additional study physician were involved. For one patient data set, no consensus on the triage category could be achieved. Thus, for this patient, the triage categories red and black both were accepted as being correct.

Calculating the rates of over- and under-triage was performed as described by Gutsch et al. [14]. Over-triage was assumed, if a patient was assigned to a higher triage category than indicated by the standard solution (i.e. red instead of yellow, green or black; yellow instead of green). If a non-critical patient was declared red, critical over-triage was recognized. Under-triage was defined as assigning a false low triage category (i.e. yellow instead of red; green instead of yellow or red; black instead of red). For the failure to declare a critical patient as red, the term critical under-triage was used.

In addition, the time requirement for performing the triage was evaluated. Triage time was taken from arrival at the patient dummy until reaching the next patient. This time included triage, labelling of the patient according to the triage category, and walking to the subsequent patient. Since opening the airway and stopping bleeding was just indicated but not fully performed, the time need for lifesaving interventions is not adequately represented.

For analysing the influence of certain personal study participant characteristics on triage performance, pre-specified subgroups of the participating EMS providers concerning professional education, career/volunteer status and previously attended triage training were compared.

Qualification levels of the triage teams were assessed on the basis of the minimal requirements for vehicle staffing. According to the European Committee for Standardization (CEN) standard, there are mobile intensive care units (mICU; CEN 1789:2007 type C) and patient transport ambulance (CEN 1789:2007 type A). The mICU group consisted of all triage runs in which the team leader was trained as paramedic, with arbitrary qualification of the second member. Triage processes with the team leader being EMT with the same or lower education of the second team partner, were assigned to the patient transport ambulance group. As a third group, disaster response teams, defined as both team members having qualifications lower than EMT, were evaluated. Triage runs with team configurations which did not meet these criteria (e.g. team leader: EMT, triage assistant: paramedic) where excluded from this subgroup analysis.

For comparing employment status and previous triage training, only homogeneous teams were included (i.e. both team members full-time professionals vs. both volunteers; both previous training vs. both no previous training).

### Statistics

For triage performance, sensitivity, specificity, positive and negative likelihood ratios (LR), and rates for correct triage category assignment and life-saving interventions, over- and under-triage, as well as critical over- and under-triage were calculated. Times are displayed as mean, minimum/maximum, median, and 25^th^, 75^th^, 90^th^, and 95^th^ percentiles. The 95% confidence interval (CI) is reported where appropriate. Statistical comparisons were made using a one-way analysis of variances (ANOVA) with Bonferroni post-hoc testing. Significance was assumed if P < 0.05. Calculations were made using the IBM SPSS Statistics 21 software (IBM Corp., Armonk, NY, US).

Results were compared to published data of the mSTaRT evaluation on the basis of CI as published by Gutsch et al. [14].

## Results

A total of N = 780 triage sequences have been analysed.

### Study participant demographics

In six training and certification sessions performed in the year 2013, 82 EMS providers (60 professional and 22 volunteer EMS providers) were trained to perform ASAV, including the complete EMS career staff of the district. Two EMS professionals did not participate in the certification process, and two persons were excluded due to participation in the ASAV development process or in teaching. Two more staff members underwent certification, but declined study participation. Thus, a total of 76 EMS providers (55 full-time personnel and 21 volunteers) could be included into the study. From two teams, the data were incomplete due to study refusal of the team partner.

Study participant qualifications were “Rettungsassistent” (German paramedic, two years of training, N = 43), “Rettungssanitäter” (German emergency medical technician (EMT), 520 hours of training, N = 13) or a lower education (N = 20). Thirty eight test persons had participated in a previous triage training following the mSTaRT concept, for five persons it was unknown whether such a training had been attended. For further information on study participant characteristics please refer to Table 1.

**Table 1:** Study participant characteristics

	**Career personnel**	**Volunteer personnel**
**Professional Qualification**		
Rettungsassistent/Paramedic	41 (54%)	2 (3%)
Rettungssanitäter/EMT	9 (12%)	3 (4%)
Other	5 (7%)	16 (21%)
**Previous Triage Training**		
mSTaRT education	37 (49%)	3 (4%)
None	16 (21%)	15 (20%)
Unknown	2 (3%)	3 (4%)

Figures represent the number of study participants and the percentage of the total study participants (n = 76) in the respective category with respect to qualification and previous triage experience by employment status.

### Test performance criteria

Over the complete study population, 83.9% of cases were correctly classified as per the triage category. Concerning the red patients, the sensitivity was 87.4% and the specificity was 91.0%. The positive likelihood ratio was 9.7 and the negative LR was 0.139 (Table 2).

**Table 2:** Test performance criteria results by study participant characteristics

		**Professional education (staffing)**			**Employment status**		**Previous mSTaRT training**		
	**Whole collective**	**Mobile intensive care units**	**Patient transport ambulance**	**Disaster response teams**	**Career teams**	**Volunteer teams**	**Yes**	**No**	**MSTaRT [**[14]**]**
	**(n = 780)**	**(n = 420)**	**(n = 40)**	**(n = 100)**	**(n = 480)**	**(n = 140)**	**(n = 220)**	**(n = 160)**	
**Test performance criteria**									
Correct triage	83.9%	85.2%	90.0%	88.0%	84.3%	87.9%	88.6%	83.8%	84.8%
	(81.3 – 86.5)	(81.8 - 88.6)	(81.8 - 100.0)	(81.6 - 93.8)	(81.1 – 87.3)	(82.1 - 93.1)	(84.2 - 92.8)	(77.9 - 89.3)	(78.7 - 91.0)
Sensitivity	87.4%	89.2%	100.0%	90.0%	88.3%	91.3%	93.8%	86.8%	88.2%
	(83.7 – 90.9)	(84.7 - 93.6)		(81.0 - 97.9)	(83.6 – 92.5)	(84.5 - 97.2)	(88.5 - 97.8)	(78.7 - 93.9)	(73.4 - 95.3)
Specificity	91.0%	89.6%	92.3%	95.6%	90.2%	95.4%	90.7%	93.6%	93.9%
	(88.1 – 93.8)	(85.0 - 93.6)	(81.0 - 100.0)	(88.6 - 100.0)	(86.1 - 93.6)	(89.9 - 100.0)	(85.1 - 95.7)	(87.7 - 98.7)	(87.3 - 97.2)
LR +	9.7	8.6	13.0	19.1	9.01	19.8	10.1	13.6	14.5
LR -	0.139	0.121	0.000	0.105	0.130	0.091	0.068	0.141	0.126
Over-triage	6.4%	6.9%	7.5%	6.0%	6.3%	6.4%	6.8%	6.3%	8.3%
	(4.9 – 8.1)	(4.9 - 8.1)	(0.0 - 16.7)	(1.9 - 10.9)	(4.3 – 8.6)	(2.9 - 10.6)	(3.9 - 10.2)	(2.7 - 10.6)	(3.6 - 13.0)
Critical over-triage	4.4%	4.4%	5.0%	2.0%	4.8%	2.1%	4.5%	3.1%	5.3%
	(3.0 – 5.8)	(3.0 - 5.8)	(0.0 - 13.5)	(0.0 - 5.1)	(3.0 – 6.8)	(0.0 - 4.8)	(2.2 - 7.5)	(0.6 -6.2)	(1.5 - 9.1)
Under-triage	9.7%	9.7%	2.5%	6.0%	9.4%	5.7%	4.5%*	10.0%*	6.8%
	(7.7 – 11.8)	(7.7 - 11.8)	(0.0 - 8.3)	(1.9 - 10.7)	(6.9 – 12.2)	(2.2 - 10.1)	(2.0 - 7.5)	(5.6 - 14.9)	(2.5 - 11.1)
Critical under-triage	4.8%	4.8%	0.0%	5.0	4.2%	2.1%	2.7%	6.3%	3.0%
	(3.3 – 6.3)	(3.0 - 4.8)		(1.0 - 9.4)	(2.4 – 6.2)	(1.4 - 7.9)	(0.9 - 5.0)	(2.9 - 10.1)	(0.1 - 6.0)
Bleeding control	93.1%	92.4%	82.5%	93.0%	90.8%	92.1%	89.5%†	93.8%†	-
	(91.3 - 94.7)	(89.7 - 94.8)	(69.4 - 93.8)	(87.4 - 97.6)	(88.0 - 93.3)	(87.3 - 96.1)	(85.1 - 93.1)	(89.7 - 97.4)	
Airway opening	90.0%	82.9%	85.0%	84.0%	82.4%	83.6%	80.0%	83.1%	-
	(87.9 - 92.1)	(79.4 - 86.3)	(72.7 - 95.6)	(76.5 – 90.9)	(79.0 - 85.5)	(77.3 - 89.4)	(74.4 - 85.2)	(77.3 - 89.0)	

Values in parenthesis represent the 95% confidence interval. *,†: P < 0.05 (ANOVA).

In 6.4%, the patients were over-triaged, and 4.4% critically over-triaged. In 9.7% under-triage was noted, which was critical in 4.8% (Table 2).

Decisions on providing life saving measures (airway opening or bleeding control) were indicated properly in 90.0% and 93.1%, respectively (Table 2).

In a subgroup analysis, triage performance of typical crew compositions of mICU (paramedic + paramedic or lower education), patient transport ambulance (EMT + EMT or lower education), and disaster response personnel (other than paramedic or EMT) were compared. No significant differences could be found with regard to the test performance criteria. However, there was a tendency towards better likelihood ratios with decreasing formal crew competency (Table 2).

Previous mSTaRT training was associated with a significantly lower rate of under-triage (4.5 vs. 10.0%, P = 0.036), but a slightly weaker decision making on bleeding control (89.5 vs. 93.8, P = 0.043) (Table 2).

The comparison of career vs. volunteer personnel revealed no significant differences concerning the test performance criteria.

### Time requirements

In average, it took a trainee 35.4 sec (95% CI 34.2 - 36.4 sec) to perform the assessment process. Disaster response teams acted slower than those of patient transport ambulances (39.1 vs. 30.6 sec, P = 0.029). Compared to mICU staff, there was an insignificant trend favouring the latter (39.1 sec for disaster response teams vs. 35.0 sec, P = 0.071). No difference could be observed between mICU and ambulance staffing (Table 3).

**Table 3:** Time requirement results by study participant characteristics and comparison to mSTaRT

		**Professional education (staffing)**			**Employment status**		**Previous mSTaRT training**		
	**Whole collective**	**Mobile intensive care units**	**Patient transport ambulance**	**Disaster response teams**	**Career teams**	**Volunteer teams**	**Yes**	**No**	**MSTaRT [**[14]**]**
	**(n = 780)**	**(n = 420)**	**(n = 40)**	**(n = 100)**	**(n = 480)**	**(n = 140)**	**(n = 220)**	**(n = 160)**	
**Time requirement in seconds**									
Average	35.4	35.0	30.6*	39.1*	34.6†	38.4†	33.6	36.5	41.0
	34.2 - 36.4	33.3 - 36.6	25.9 - 35.3	35.4 - 43.0	33.1 - 36.1	35.5 - 41.4	31.7 - 35.8	33.6 - 39.5	
Median	35.0	34.0	28.5	39.0	34.0	39.5	33.0	36.0	35.0
	34.0 - 37.0	32.0 - 36.0	25.5 - 40.0	35.0 - 43.0	32.0 - 37.0	36.0 - 43.0	31.0 - 35.0	32.0 - 39.0	
Range	3 - 104	3 - 104	5 - 55	5 - 88	3 - 104	5 - 88	4 - 76	4 - 88	10 - 121
25^th^ percentile	24.0	24.3	19.8	26.0	24.0	26.0	25.0	23.3	25.0
	(21.3 – 25.2)	(21.0 – 26.0)	(9.0 – 27.0)	(18.0 – 30.0)	(21.0 – 26.0)	(19.0 – 30.0)	(21.0 – 27.0)	(17.0 – 27.0)	
75^th^ percentile	46.0	46.0	45.0	50.0	46.0	49.8	44.0	49.0	49.0
	(45.0 – 48.0)	(43.0 – 48.0)	(35.0 – 47.0)	(45.0 – 56.0)	(32.0 – 37.0)	(45.8 – 54.0)	(41.0 – 47.0)	(45.0 – 53.8)	
90^th^ percentile	57.9	58.0	49.9	65.8	56.0	58.9	53.0	62.0	65.0
	(55.0 – 61.0)	(53.0 – 62.0)	(45.0 – 54.5)	(56.0 – 75.2)	(53.0 – 60.8)	(55.7 – 67.5)	(50.0 – 59.0)	(56.0 – 69.0)	
95^th^ percentile	66.0	67.0	53.0	76.0	65.0	70.0	60.0	70.0	98.0
	(63.0 – 69.9)	(62.0 – 70.0)	(47.0 – 55.0)	(64.7 – 86.0)	(61.0 – 69.0)	(60.4 – 77.6)	(55.0 – 67.0)	(64.5 – 77.0)	

Values in parenthesis represent the 95% confidence interval. *, †: P < 0.05 (ANOVA).

Consistently, volunteer personnel needed more time for triaging than professional staff (38.4 vs. 34.6 sec, P = 0.024). Previous mSTaRT training had no significant impact on triage speed (P = 0.086, Table 3).

Further, the time spent on triage was dependent on the expected triage category: red and yellow patients required the most time to be assessed, dead patients required significantly less time (P < 0.01, respectively), and triage was conducted the fastest on green patients (P < 0.01 compared to all other categories, respectively) (Table 4).

**Table 4:** Mean time requirements for primary survey, according to expected triage category

**Expected triage category**	**Mean time requirement (sec)**	**Statistical difference to**		
	**(95% confidence interval)**	**Red**	**Yellow**	**Green**
Red	41.8			
	(40.4 - 43.3)			
Yellow	38.9	n.s.		
	(37.1 - 40.7)			
Green	14.5	**	**	
	(12.7 - 16.6)			
Dead	28.8	**	**	**
	(24.4 - 34.0)			

N = 780. **P < 0.01 (ANOVA).

### Comparison to mSTaRT

Based on the comparison of CI, no significant difference for sensitivity, specificity, over- and under-triage between ASAV and mSTaRT could be detected (Table 2). Triage following the ASAV algorithm could be completed in a mean time of 35.4 sec per patient, compared to 41 sec with mSTaRT [14]. The timely advantage favouring ASAV was also present regarding the 75^th^, 90^th^, and 95^th^ percentile (Table 3). Since no CI are published for mSTaRT time requirements, a conclusion on statistical significance of these differences cannot be drawn.

## Discussion

MCI management aims towards priorisation of severely injured or critically ill during assessment, treatment and transport. Regarding trauma, the patient should be treated in an appropriate trauma centre within one hour from incident onset [15], even in an MCI setting. This necessitates immediate, correct and complete identification of “red” patients by paramedics as early as possible, no matter whether the chief emergency physician in the field has already arrived at the scene for a secondary, physician based triage. For this purpose, numerous standardised approaches have been developed, but none has become widely accepted on an evidence basis [10]. Until now, no such algorithm is specifically designed for a physician based EMS system. With the implementation of ASAV, the authors sought to fill this gap. ASAV is designed for primary triage by paramedics, which is mandatorily followed by a secondary triage by a pre-hospital emergency physician or chief emergency physician in the field. Furthermore, ASAV is in accordance with the requirements of the German triage consensus conference from 2012 [16], and most of the core criteria on primary triage, as defined by a US national guideline on MCI triage [10]. In this study, we aimed to evaluate the reliability and time requirements of the ASAV concept with a special scope on the identification of the critical patients.

Accepted requirements for primary triage algorithms are simplicity [16],[10],[12], and to refrain from time consuming counting the respiratory frequency during primary triage [10]. Both aspects have been addressed in ASAV by reducing decision steps concerning respiration to a single one, and replacing respiratory frequency by a short list of distinct breathing disorders, which are not based on measured values.

### ASAV test performance

According to the collected data, the ASAV triage led to a correct triage classification in 84%, with sensitivity and specificity for red patients of around 90%. Decisions on lifesaving interventions were made with an accuracy of greater than 90%. The ASAV test performance is comparable to that of other algorithms used for the same purpose, but might consume less time.

Since on one patient data set no consensus concerning the standard solution could be achieved, in this special case both the red and the black category were accepted as being correct. In general, only patients with apparent deadly injuries are expected to be sieved in the black category. In doubt, or in the absence of definite signs of death, such as decapitation or destruction of the torso, EMS personnel are expected to classify the patients as “red”. Most concepts for primary triage, as well as the US guideline [10], require the black category for apnoeic patients and those severely injured with an estimated hopeless prognosis under the given circumstances, even if definite signs of death are absent. In a physician based EMS system, paramedics have no allowance to pronounce death and or to withhold lifesaving therapy from critical patients during MCI. As a consequence, in ASAV all patients with impaired vital functions, which are lacking definite signs of death, are classified in the red category. This is in accordance with the recommendations of the German triage consensus conference of 2012 [16], and meanwhile has been adopted by other triage concepts, too [17].

### Subgroup analysis

According to the German Medical Association [18] preliminary triage is requiring the paramedic qualification. This regulation might lead to a delay in primary triage in case of a large MCI in a remote area with limited EMS resources, since the presence of a sufficient number of paramedics takes a considerable amount of time, while personnel with other professional education might be readily available. Our results suggest that ASAV triage performance might be independent of provider qualification or employment relationship, and thus, could be equally reliable deployed by trained EMTs or volunteer disaster response units. However, whether the necessary evaluation of the vital parameters can be achieved in a valid way by personnel with varying training levels, needs to be addressed separately.

Some study participants attended mSTaRT training within 12 months prior to this study. Those persons achieved better triage results in only one parameter (under-triage) than previously untrained ones (Table 2). This marginal difference might be interpreted as a hint that triage algorithm training needs to be repeated after a short interval. To answer this question, further studies are needed. The fact that mSTaRT experienced study participants performed weaker in regard to bleeding control decisions had no obvious explanation, since both concepts include literally the same indication (“spurting haemorrhage”) [19],[12].

### Time requirement

In this study, ASAV triage required a little more than half a minute per patient. As demonstrated, the time needed for triage is dependent on the triage category. Thus, in real world scenarios, the duration of the primary triage will differ according to the distribution pattern of injury severity. Our data, on the other hand, can be helpful for incidence planning. Assuming a patient distribution of 20% red patients, 20% yellow, and 60% green cases [16], an average triage duration of around 25 sec per patient could be calculated from the data in Table 4.

The finding that less experienced medical personnel needs more time to perform triage can be explained by volunteers being more hesitant to decide on their triage results, while the professionals required less time to translate the written patient characteristics into triage decisions, as it was noticed during the course of the study.

### Comparison to mSTaRT

The presumably most established algorithm in Germany, mSTART, has been evaluated in 2006 regarding its reliability [14]. After the training of 244 paramedics in the application of mSTaRT, 22 thereof triaged a total of 132 patients during the course of three MCI exercises. As in the present study, sensitivity, specificity, and further test performance parameters where calculated from the comparison of actual triage results and a standard solution. According to the comparison of confidence intervals, no relevant or significant differences to the reliability of ASAV could be detected.

As an effect of replacing the respiratory frequency by a criterion based more on the clinical presentation (“breathing difficulties”) than on counting a single vital sign, the mean time needed to perform primary triage by ASAV is 35 sec (95% CI 34.2 - 36.4) compared to 41 sec with mSTaRT [14]. This time advantage is observed, although the time span for ASAV evaluation included tagging (fixing marker bands at the patient) and walking to the next victim. Both steps were excluded from measured times in the work of Gutsch and co-workers.

### Merits and limitations of the study

The usage of the patient dummies, which were randomly distributed to training and evaluation sessions, ensures that all subjects where trained and tested with the same reproducible set of "patients", which is not depending on the potentially varying behaviour of patient actors. A disadvantage of the dummy use is that clinical patient conditions cannot be mimicked. Thus, the value of the study is limited to the formal application of the algorithm, and lacks evidence on the ability to assess patients clinically. In addition, the scope of the study is laid on the reliability of the algorithm, and, since no real patient data was evaluated, no conclusions on its validity can be drawn.

An important prerequisite for ensuring compliance with the algorithm is its provision as a written document during the triage process. If in real world situations the algorithm is not available, it is most likely that the results of this study cannot be extrapolated. Thus, in the EMS district of the authors, the written algorithm is contained in an MCI package which is held available on every mICU.

A triage procedure which rigidly follows an algorithm such as ASAV is not suitable to be dynamically adapted to varying circumstances of different (and possibly dynamically changing) MCI scenarios. Thus, ASAV is designed for preliminary primary triage, but not for definite re-triage or secondary triage. In addition, we strongly recommend that in physician manned EMS systems each patient is re-triaged at the scene by an experienced emergency physician.

With 780 evaluated triage runs, to the best of our knowledge, the present study is the largest prospective evaluation of a triage algorithm in the literature. This resulted in sufficient subgroup size, and analysis concerning professional qualification, employment status, and previous triage training were facilitated.

By including almost the complete professional staff from a district with six ambulance stations, a selection bias concerning the study participants can nearly be ruled out. On the other hand, the comparison between volunteers and career staff is limited by the fact that only a single volunteer organisation was included into the study. Therefore, the volunteer study population might not be representative.

Concerning the comparison with mSTaRT, there are differences in study designs. Most prominently, the use of dummies in a teaching environment (ASAV) in comparison to the evaluation of patient actors in the context of several full-scale exercise scenarios (mSTaRT) with the above discussed advantages and disadvantages needs to be put forward.

## Conclusion

The ASAV is a concept specifically for primary triage by non-physician EMS personnel in physician based EMS systems. ASAV reliably detects red patients. The test performance criteria are comparable to that of mSTaRT, whereas ASAV triage might be accomplished faster. We found no hints, that EMTs or volunteer disaster response teams were performing primary triage using the ASAV approach with lesser reliability. Further studies are needed to address the validity of either triage concept.

## Abbreviations

ANOVA: Analysis of variances

ASAV: Amberg-Schwandorf algorithm for primary triage (Amberg-Schwandorf-Algorithmus für die Vorsichtung)

CEN: Comité Européen de Normalisation (European Committee for Standardization)

CI: Confidence interval

EMS: Emergency medical services

EMT: Emergency medical technician

LR: Likelihood ratio

MCI: Mass casualty incident

mICU: Mobile intensive care unit

mSTaRT: Modified Simple Triage and Rapid Treatment

## Competing interest

The authors declare that they have no competing interests.

## Authors’ contributions

PW participated in study design, data acquisition, data analysis, and manuscript preparation. MB participated in study design, data acquisition, and manuscript preparation. BMG participated in study design, and manuscript preparation. TB participated in study design, data analysis, and manuscript preparation. MSD was responsible for overall scientific coordination and participated in study design, data acquisition, data analysis, and manuscript preparation. All authors read and approved the final manuscript.
